# Awareness and level of digital literacy among students receiving health-based education

**DOI:** 10.1186/s12909-024-05025-w

**Published:** 2024-01-08

**Authors:** Alp Aydınlar, Arda Mavi, Ece Kütükçü, Elçim Elgün Kırımlı, Deniz Alış, Ata Akın, Levent Altıntaş

**Affiliations:** 1 Enka High School, Istanbul, Türkiye; 2 Acibadem University School of Medicine, Istanbul, Türkiye; 3 Faculty of Engineering and Natural Sciences, Acibadem University, Istanbul, Türkiye; 4 Department of Radiology, Acibadem University School of Medicine, Istanbul, Türkiye; 5 Department of Basic Sciences, Acibadem University School of Medicine, Istanbul, Türkiye

**Keywords:** Digital literacy, Health, Students, University, Education

## Abstract

**Background:**

Being digitally literate allows health-based science students to access reliable, up-to-date information efficiently and expands the capacity for continuous learning. Digital literacy facilitates effective communication and collaboration among other healthcare providers. It helps to navigate the ethical implications of using digital technologies and aids the use of digital tools in managing healthcare processes. Our aim in this study is to determine the digital literacy level and awareness of our students receiving health-based education in our university and to pave the way for supporting the current curriculum with courses on digital literacy when necessary.

**Method:**

Students from Acibadem University who were registered undergraduate education for at least four years of health-based education, School of Medicine, Nutrition and Dietetics, Nursing, Physiotherapy and Rehabilitation, Psychology, Biomedical Engineering, Molecular Biology, and Genetics were included. The questionnaire consisted of 24 queries evaluating digital literacy in 7 fields: software and multimedia, hardware and technical problem solving, network and communication/collaboration, ethics, security, artificial intelligence (A.I.), and interest/knowledge. Two student groups representing all departments were invited for interviews according to the Delphi method.

**Results:**

The survey was completed by 476 students. Female students had less computer knowledge and previous coding education. Spearman correlation test showed that there were weak positive correlations between the years and the “software and multimedia,” “ethics,” “interest and knowledge” domains, and the average score. The students from Nursing scored lowest in the query after those from the Nutrition and Dietetics department. The highest scores were obtained by Biomedical Engineering students, followed by the School of Medicine. Participants scored the highest in “network” and “A.I.” and lowest in “interest-knowledge” domains.

**Conclusion:**

It is necessary to define the level of computer skills who start health-based education and shape the curriculum by determining which domains are weak. Creating an educational environment that fosters females’ digital knowledge is recommended. Elective courses across faculties may be offered to enable students to progress and discuss various digital literacy topics. The extent to which students benefit from the digital literacy-supported curriculum may be evaluated. Thus, health-based university students are encouraged to acquire the computer skills required by today’s clinical settings.

**Registration:**

This study was approved by Acıbadem University and Acıbadem Healthcare Institutions Medical Research Ethics Committee (ATADEK) (11 November 2022, ATADEK registration: 2022-17-138) All methods were carried out in accordance with relevant guidelines and regulations. Informed consent was obtained from the participants.

**Supplementary Information:**

The online version contains supplementary material available at 10.1186/s12909-024-05025-w.

Infrastructures used in health are rapidly digitalizing, significantly improving the quality of diagnosis, follow-up, and treatment processes of patients [1]. Digital literacy is essential for health science students to access and interpret data, analyze medical images, and use technology to improve patient care [2]. Additionally, digital literacy plays an essential role for health science students to understand the ethical implications of using technology in healthcare and its potential risks [3]. In the Future Health Index 2020 report, it is stated that the presence of medical technologies motivates especially young health workers and that proper training should be provided to this generation on the correct use and interpretation of technology and data [4]. In their research conducted by de La Hoz et al. on bioscientific university students and especially evaluating eHealth literacy (digital literacy in health), they concluded that university education programs applied to develop digital and scientific skills in health education should be improved to meet future needs [5]. Digital health was incorporated as an elective subject in an integrated medical degree program for a study in Hamburg, Germany. Consequently, in a poll evaluating the courses presented in the given semester, this class received the highest overall satisfaction, reflecting students’ general fulfillment with digital health education [6]. In another study in Germany, distinct modules with learning objectives were created for each academic year of medical school to teach digital competencies, focusing on digital challenges related to the relevant medical application fields. Given the rapid digitalization of medicine, they underlined the importance of teaching digital literacy in undergraduate medical education [7].

In general, 21st-century students are compelled to be proficient digital natives. However, the digital literacy skills and competencies they already possess when they start university do not always mean they can use them purposefully to support their learning [8]. Students use digital tools for many services, including social and educational activities [9]; however, their competencies in primary digital tools such as Microsoft Office, Word, PowerPoint, and Excel fall short of expectations [10]. A study from Japan revealed that although there is 100% smartphone and personal computer ownership among that first-year university students, these devices are not used in any depth [11]. Simon et al. noticed a need to develop and implement training programs for the curriculum to acquire digital knowledge. They suggest that identifying diverse levels of digital literacy among students, assessing professors’ abilities to incorporate literacy aspects into their teaching methods, and executing a plan to enhance digital literacy across curriculum disciplines will lay the foundations for a more robust digital capacity in students [12]. Most importantly, the faculty’s digital knowledge should be assessed and strengthened to achieve higher digital proficiency in the university environment. Sánchez-Cruzado et al. showed that the competency levels of the teachers were at a low intermediate level (B1 level), and only 1.7% of the faculty surveyed reached a high level [13].

In a period where artificial intelligence (A.I) has entered many areas, it is essential to guarantee that students studying health sciences, have a general foundation of A.I. literacy. A.I. should be introduced to their education, given this technology’s significance and potential impact [14]. ChatGPT is one of the most commonly used A.I. tools and is an effective amplifier in medical education with increasing knowledge interpretation, learning, and recall. It is seen as a practical online teaching assistant due to its instant responses and online availability [15]. Sensitive information, including patient data, is frequently shared between students, instructors, and medical professionals during medical practice. Transferring this information with ChatGPT is questionable from an ethical standpoint, and the user must ensure sufficient precautions to keep this information private [16]. Healthcare students’ levels of digital literacy should be evaluated in that manner, and no compromises should be made since AI technologies may play increasingly important roles in their jobs and research in the future.

Our aim in this study is to determine the digital literacy level and awareness of our students receiving health-based education in our university and to pave the way for supporting the current curriculum with courses on digital literacy, when necessary.

## Method

### Participants

Students who has been receiving undergraduate education for at least four years of health-based education at the same university were included. These students were recruited from the faculties of the School of Medicine, Health Sciences (Nutrition and Dietetics, Nursing, Physiotherapy, and Rehabilitation), Engineering and Natural Sciences (Biomedical Engineering, Molecular Biology, and Genetics), and Humanities and Social Sciences (Psychology). The same survey was also applied to Computer Engineering students who acquired extensive training in digital literacy but did not receive any health-based education. The main purpose was to compare Computer Engineering students’ scores obtained from the questionnaire and interview with those who are receiving health-based education. Thirty-nine computer engineering students agreed to participate in this study. It should be noted that, Computer Engineering students were excluded from the sociodemographic data.

### Assessment

#### Instrument

We developed a questionnaire of 24 queries evaluating digital literacy in 7 fields: software and multimedia, hardware and technical problem solving, network and communication/collaboration, ethics, security, artificial intelligence, and interest-knowledge (Table 1). While creating this scale, we also included the questions from the previously published digital literacy scale developed by Ng W [17], adapted in Turkish [18]. This 10 items scale evaluates digital literacy in 3 main aspects: technical, cognitive, and socioemotional. Students were asked to enter their sociodemographic information and basic view on digital literacy at the start of the questionnaire. The responses were given on a 5-point Likert scale: “I strongly disagree,” “I disagree,” “Neutral,” “I agree,” and “I strongly agree.” The questionnaire was reviewed by faculty from engineering, statistics, and medicine backgrounds and was e-mailed to the participants.

**Table 1:** Questionnaire evaluating digital literacy in 7 domains

Domain	Questions
Software and Multimedia	1.I can learn new technologies easily. 2.I have the technical skills to use information and communication technologies for learning purposes and to develop digital teaching materials (For example, presentations, digital stories, wikis, and blogs) to showcase what I have learned. 3.I can easily communicate with others through platforms such as Zoom, Microsoft Meeting, and Outlook. 4.I know computer languages ​​(such as Python, C++, and visual basic).
Hardware	5.I know how to solve technical problems I encounter with the technologies I use. 6.I know the essential parts of the computer (such as motherboard, memory, computer case, power supply, and hard disk), and their functions. 7.I know and can use wireless communication modules such as wifi and Bluetooth.
Network	8.I am confident in my searches and evaluations to obtain information on the Internet. 9.Information and communication technologies allow me to better collaborate with my peers on working on a project and other learning activities. 10.I often help out with my friends over the internet in my studies at University (e.g., via Skype, Facebook, and Blogs). 11.I use professional media platforms such as LinkedIn to take steps toward my career goals and to find or reach people related to my profession.
Ethics	12.I do not get stolen information from other works during my research and presentations. I cite. 13.I have an idea about digital copyrights and licenses. 14.I am aware of the Personal Data Protection Law. 15.I am aware of the Ethics Committee processes and conditions that control under which conditions and with whom I can share consented patient information and data.
Security	16.I am familiar with topics related to internet activities, such as cyber security, web search, and internet fraud. 17.I know how the information I enter on the technological devices and applications I use is stored, and I am aware of who can see and use it.
AI	18.I am aware that artificial intelligence is included in the technologies we use in our daily lives. 19.I am aware that there are lectures in our University on integrating artificial intelligence into the medical field. 20.I know the areas where artificial intelligence is used in medicine.
Interest-Knowledge	21.I follow important new technologies. 22.I am familiar with many different technologies. 23.The skills I have in information and communication technologies are sufficient. 24.I use the Internet to access medical information and check the reliability of sources.

#### Interview

Experts among the researchers prepared four open-ended questions for the participants who had already completed the questionnaire. The queries were revised after a pilot interview with a small group of students. Two groups were determined and selected by a simple random method, structured as one student from each field, adding up to eight participants. Students were drawn from a waiting list when the assigned participant could not attend.

The questions were as follows:

1. The European Commission has defined digital literacy as the ability to use information and communication technologies and the internet for creativity, innovation, and entrepreneurship and to acquire the knowledge and skills necessary to live in the 21st century [19]. Some of these digital literacy skills are using computers, smartphones, tablets, networks, and software (e.g., searching the internet, utilizing Word and Excel, reading e-mails, making various settings on the device) and installing/deleting programs.

Do you think that you have sufficient knowledge and skills in digital literacy?

2) New digital technologies in the health field mainly consist of artificial intelligence-based decision support software, robotic surgery, the production of personalized prostheses with 3D printing methods, telemedicine, chatbot, and teleconsultation.

What benefits and challenges will they bring in the future?

3) Do you feel competent about using and developing new technologies? Why?
4) Do you feel adequately equipped regarding data security, ethical use, and sharing by introducing these technologies into our lives? (such as Personal Data Protection Authority) Why?

Zoom platform was used to record the virtual semi-structured meeting. After the participant’s information and verbal consent were taken, the assigned interviewer directed each question written on a PowerPoint presentation to each participating student. The interviewer did not comment or provide additional information to explain the question but added short queries to make the conversations more productive.

The research group faculty transcribed the interview for qualitative analysis, and relevant keywords were identified and themed according to the Delphi method [20].

### The Delphi method

The Delphi method is a methodical procedure that uses the panel members’ pooled judgment. This method collects opinions on a particular research question to gain agreement. The panel of participants can review and reflect on their thoughts in light of the input from other peers’ viewpoints.

The Delphi method is helpful in polemical areas where statistical model-based data still needs improvement and where experts need more consensus. Usually, there are two rounds of questions in Delphi research.

### Statistics

All statistical analyses were conducted in R environment v4.3.0 [21] with RStudio software v2023.03.0 [22]. Graphics and tables were prepared in Microsoft Excel 2019 [23]. Statistical tests with *p*-values less than 0.05 and an alpha 0.05 (two-sided) were considered significant results.

Data were summarized as mean, standard deviation (S.D.), median and interquartile region (IQR), and the number of data points and percentages. The answers to each question were on a 5-point Likert scale, and for each participant, the scores of the questions related to that domain were averaged. A final score was calculated as the mean score of all fields.

Shapiro-Wilk Normality Test was used to confirm the normality of data distribution for statistical tests. The Scores were compared between groups with a t-test and Mann-Whitney U test. The correlation analyses were carried out with Spearman’s Rank Correlation method.

## Results

### Demographics

Four hundred seventy-six (476) university students studying in 7 academic disciplines of health-related sciences at the same university participated in the study. The students were comprised of 77.10% females and 22.90% males. The Nursing department accounted for 43.07% of the participants, while the participation rates in the other six departments varied, ranging from 5.75 to 12.94%. The study involved individuals hailing from six academic years. Year 5 and 6 students were studying in the School of Medicine, which fills about 10% of the subjects. The highest number of students were in year 1 (31.72%). The other students were evenly distributed between years 2–4.

Most participants (71.22%) believed digital literacy education was essential for a health-based student. In comparison, 25.63% stated that digital literacy training was necessary but optional for a health-based student, and only 15 students (3.15%) thought digital literacy education was unnecessary.

Almost 63% of participants had never received any type of coding programming/coding education. 15.76% learned coding at university, 17.22% were already exposed to any coding training before university, and 3.78% have learned to code independently (Table 2).

**Table 2:** Demographic features of 476 participants

Features	Subclass	n	%
Gender	Female	367	77.10
	Male	109	22.90
Nutrition and Dietetics		40	8.21
Physiotherapy and Rehabilitation		31	6.37
Nursing		205	42.09
Molecular Biology and Genetics		58	11.91
Psychology		28	5.75
School of Medicine		62	12.73
Biomedical Engineering		63	12.94
Year	1	151	31.72
	2	91	19.12
	3	96	20.17
	4	90	18.91
	5	33	6.93
	6	15	3.15
Initial Coding Education	No education	301	63.24
	Primary	11	2.31
	Middle	27	5.67
	High	44	9.24
	University	75	15.76
	Self-learned	18	3.78
View on Digital Literacy Education	Not Necessary	15	3.15
	Necessary but Not Essential in Health Based Education	122	25.63
	Necessary and Essential in Health Based Education	339	71.22

The exposure to coding education differed significantly between genders. The percentage of females (67.85%) who did not take any coding education at all was significantly higher than males (47.71%) (*p*: 0.0002). The percentage of males starting coding at high school and the university was higher than females (*p*: 0.0411 and 0.0127, respectively). There was no significant difference between self-learned proportions between genders. Also, no notable difference was found between genders in their views of digital literacy education in health-related departments (*p* values > 0.88) (Table 3).

**Table 3:** Proportion tests between genders

	Subcategories	*p*-value	Female %	Male %	Female (n)	Male (n)
İnitial coding education	No education	0.0002***	67.85	47.71	249 / 367	52 / 109
	Primary	0.4595	2.72	0.92	10 / 367	1 / 109
	Middle	1	5.72	5.50	21 / 367	6 / 109
	High	0.0411*	7.63	14.68	28 / 367	16 / 109
	University	0.0127*	13.36	23.85	49 / 367	26 / 109
	Self-learned	0.0534	2.72	7.34	10 / 367	8 / 109
View on digital literacy education	Not Necessary	1	3.27	2.75	12 / 367	3 / 109
	Necessary but Not Essential in Health Based Education	0.8881	25.34	26.61	93 / 367	29 / 109
	Necessary and Essential in Health Based Education	0.9754	71.39	70.64	262 / 367	77 / 109

*95%, **99%, ***99.9% confidence level for statistically significant differences in proportions of genders

### Scores

#### Comparisons of domains in digital literacy

The mean scores in each field of digital literacy lay between 3 and 4 on a 5-point Likert scale. The “network” and “A.I.” scores were the highest (mean 3.87 ± 0.61 and 3.82 ± 0.66, respectively), and the “interest-knowledge” scores were the lowest at a mean of 3.49 ± 0.66.

When scores were compared between genders, there were no considerable differences in the “network,” “ethics,” and “A.I.” areas. In contrast, male scores were significantly higher in the “software and multimedia,” “hardware,” “security,” and “interest-knowledge” domains and total scores. The most apparent difference between median scores of genders was observed in the “security” area, where the difference was 0.50 points out of a 1–5 scale (*p*: 0.0288). (Table 4).

**Table 4:** Comparison of scores between genders

Field of digital literacy	*p*-value	Female Mean (SD)	Male Mean (SD)	Female Median (IQR)	Male Median (IQR)	All Data Mean (SD)	All Data Median (IQR)
Software and Multimedia	0.0006***	3.47 (0.60)	3.69 (0.65)	3.50 (3.00; 3.75)	3.75 (3.25; 4.00)	3.52 (0.62)	3.50 (3.25; 3.25)
Hardware	< 0.0001***	3.50 (0.62)	4.02 (0.66)	3.67 (3.00; 4.00)	4.00 (3.67; 4.33)	3.62 (0.66)	3.67 (3.33; 3.33)
Network	0.7848	3.88 (0.61)	3.86 (0.60)	4.00 (3.50; 4.25)	4.00 (3.50; 4.25)	3.87 (0.61)	4.00 (3.50; 3.50)
Ethics	0.2661	3.73 (0.63)	3.79 (0.78)	3.75 (3.25; 4.00)	4.00 (3.25; 4.25)	3.74 (0.67)	3.75 (3.25; 3.25)
Security	0.0288*	3.59 (0.77)	3.79 (0.81)	3.50 (3.00; 4.00)	4.00 (3.50; 4.00)	3.63 (0.78)	3.50 (3.00; 3.00)
AI	0.4603	3.82 (0.64)	3.79 (0.71)	4.00 (3.33; 4.33)	3.67 (3.33; 4.33)	3.82 (0.66)	4.00 (3.33; 3.33)
Interest-Knowledge	0.0002***	3.42 (0.64)	3.72 (0.69)	3.50 (3.00; 3.75)	3.75 (3.25; 4.25)	3.49 (0.66)	3.50 (3.00; 3.00)
Average Score	0.0011**	3.63 (0.48)	3.81 (0.52)	3.63 (3.33; 3.92)	3.79 (3.51; 4.12)	3.67 (0.50)	3.65 (3.39; 3.39)

*95%, **99%, ***99.9% confidence level for statistically significant differences between the scores of genders

**Fig. 1:**
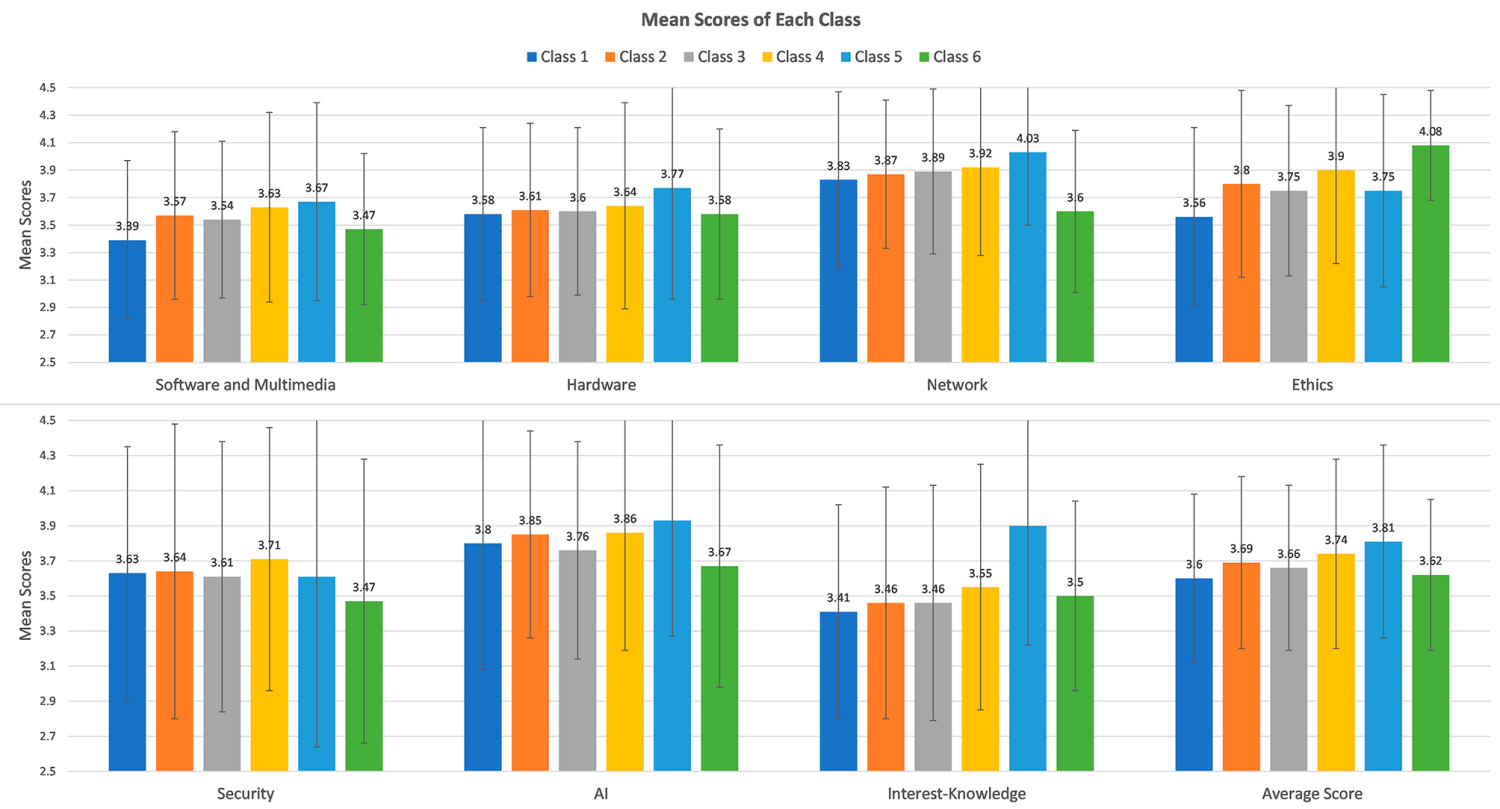
Comparison of mean scores in each domain according to years

Spearman correlation test showed that there were weak positive correlations between the years and the “software and multimedia”(*p*: 0.0008, rho: 0.1539), “ethics” (*p*: 0.0001, rho: 0.1830), “interest and knowledge” (*p*: 0.0041, rho: 0.1315) domains and the average score (*p*: 0.0230, rho: 0.1046). No significant correlation was detected between the years and the “hardware,” “network,” “security,” and “A.I.” fields (*p* values > 0.1865) (Supplementary Table 1). Figure 1 shows the mean scores and standard deviations of each year in all fields (Fig. 1).

The “software and multimedia” scores were lowest in year 1 (3.39 ± 0.58), compared to year 2 (3.57 ± 0.61), year 3 (3.54 ± 0.57), year 4 (3.63 ± 0.69) and year 5 (3.67 ± 0.72) scores (*p* < 0.020). No significant difference was found between years regarding “hardware,” “security,” and “A.I.” areas. Year 6 (3.6 ± 0.59) scored lower than year 4 (3.92 ± 0.64) and year 5 (4.03 ± 0.53) (*p*: 0.0440 and 0.0233 respectively) for the domain “network.”

In “ethics,” the lowest score was obtained in year 1 (3.56 ± 0.65), relatively lower than all years with *p*-values lower than 0.0202 excluding year 5 (3.75 ± 0.70). For the same domain, year 6 (4.08 ± 0.40) was also found to be significantly higher than year 3 (3.75 ± 0.62) and 5 (3.75 ± 0.70) (*p*: 0.0098 and 0.0420, respectively).

The field “interest and knowledge” reached the highest score in year 5 students compared to other years (3.90 ± 0.68, *p*-values < 0.0021).

The only significant difference in overall scores was observed between year 1 and 4 (*p*: 0.0102). Year 4 score (3.74 ± 0.54) was about 4% higher than year 1 (Data not shown).

In order to find out how much the scores of each department deviated from the general trend, the scores of each section were compared with the total scores of the participants from the other departments.

The scores of the Molecular Biology and Genetics, Physiotherapy, Rehabilitation, and Psychology departments did not differ from the general outlook of all scores. Biomedical Engineering displayed the highest scores in all fields except “ethics,” with at least a 6% difference. School of Medicine scores was ranked second and showed higher scores in “hardware” and “ethics” by more than 4% and in “interest-knowledge” by 8.7%.

**Fig. 2:**
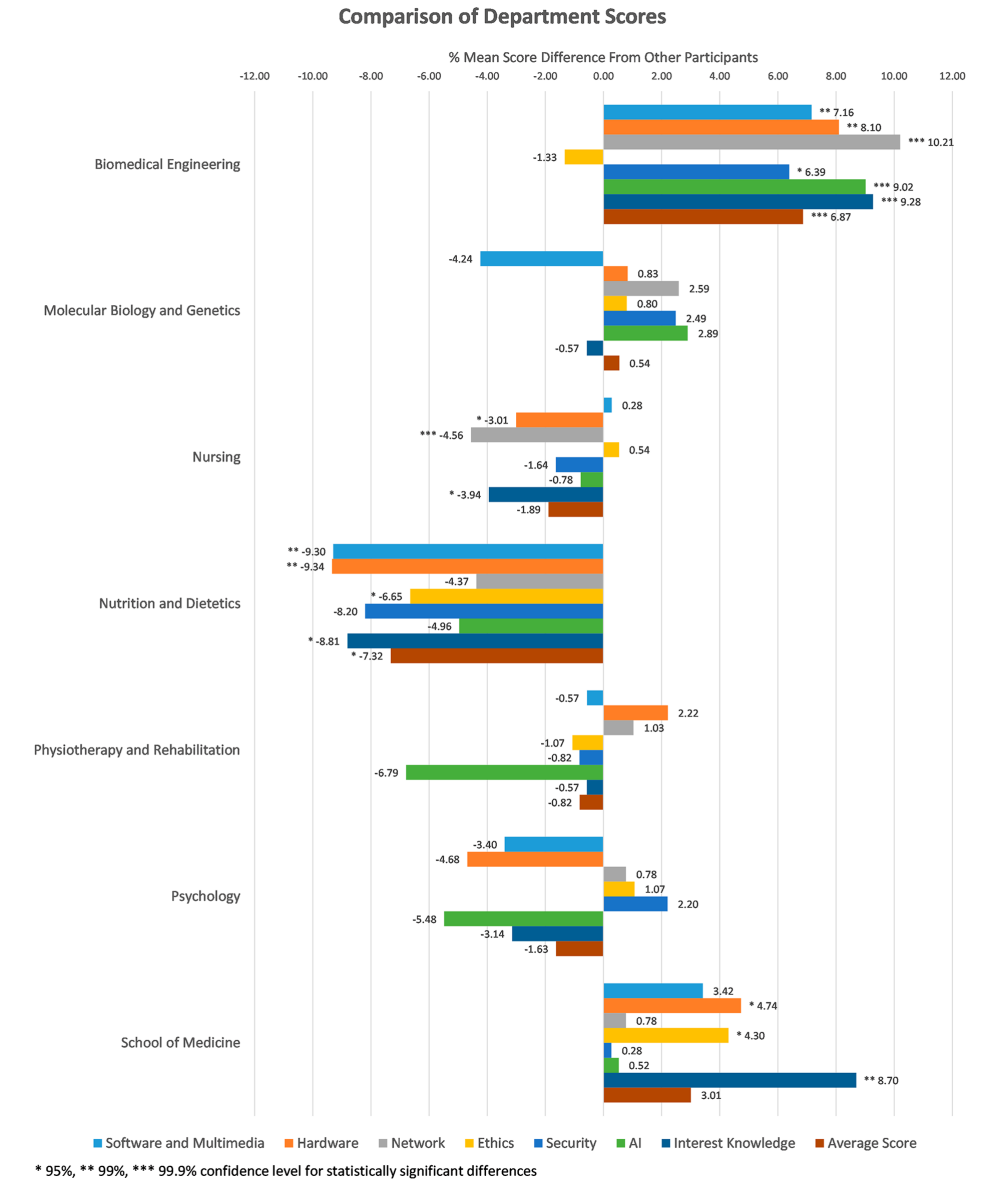
Deviation of mean scores of each domain of the departments from the general trend

The Department of Nutrition and Dietetics scores were remarkably lower than the others, showing a score difference between 6.65% and 9.34% in all domains except “network,” “security,” and “A.I.” Even in these fields, the scores were lower but not statistically significant. The second lowest scores were obtained from the Department of Nursing in “hardware,” “interest-knowledge,” and “network,” with − 3.01%, -3.94%, -4.56% difference, respectively. No statistically significant difference was found in other areas (Fig. 2).

The same questionnaire was also directed to Computer Engineering students at the same university, who were trained as the best equipped in digital literacy. The Computer Engineering students exhibited very high scores in the “software and multimedia,” “hardware,” and “security” fields (*p*-values < 0.006). In contrast, computer engineers got surprisingly ~ 11% lower mean scores than the rest of the participants in ethics. Further, their “interest and knowledge”, and “network” scores ranked second after biomedical engineering students’ scores. Interestingly, computer engineering students did not see themselves as competent in “artificial intelligence” as nursing, medical, molecular biology and genetics, and biomedical students. (Supplementary Tables 2 and Supplementary Fig. 1).

### Interview

The first interview lasted 1:22:27 h, and the second interview 1:31:32 h. All participants completed the first interview, and two dropouts were in the second. Table 5 shows the themes, subthemes, and example quotes of the participants of both interviews.

**Table 5:** Thematic Analysis

Theme	Subtheme	Example Quote
Digital Literacy	Competence and improvement needs	*Having digital resources and being able to use them is not sufficient to be digitally literate. We need to have the ability to use, understand and produce information. (P.7)* *I think I have sufficient knowledge and skills in this regard as required by my job or the responsibilities given to me. However, I am also aware that they have different uses and that some people are much more knowledgeable than me. (P.3)*
New technologies	Positive effects and results	*Robotic surgery is essential in terms of comfort and may also significantly reduce the error rate. (P.3)* *Decision support software takes data many times more than the number of patients physicians can see in their lifetime and draws conclusions that need to be learned. For this reason, this system can see things that a human cannot see. (P.3)* *Artificial intelligence-based support software, robotic surgery special prostheses, and 3D special prostheses will significantly contribute. (P.17)* *If we talk about robotic surgery in the operating room, serious errors can even be reduced to zero. (P.15)*
	Concerns	*I have a reservation that the relationship between patient and physician will turn into monotony when digitized. As I said, when such a thing happens, I think that it will be difficult to touch the patient. (P.3)* *Telemedicine will bring about as much harm and difficulty. (P.17)* *I think interactions involving human relations rather than technology are important in treating, diagnosing, or delivering patient care for most diseases. So maybe we can count this as the difficulty of new technologies. (P.15)*
	Competence and improvement needs	*I am not fully competent in terms of the use of new technologies. Let’s use it to see if we are sufficient. (P.5)* *I do not have sufficient competence in developing new technologies. But since I am open to learning about the use of new technologies, I think I can adapt quickly. (P.7)* *New technologies, especially the newest technologies, are very costly in the first place. For this reason, I can’t reach them easily in my private life or school education.(P.12)*
Artificial intelligence	Positive effects and results	*AI can be helpful in every field humans cannot reach or do. (P.10)* *Algorithms will eventually be able to make a decision at a speed that the human brain cannot. (P.14)*
	Concerns	*AI will update professions. (P.10)* *The unemployment rate may increase (P.13)*
	Competence and improvement needs	*Professions may evolve, and new careers will emerge, and as a result, we will need people who have been trained to be able to do these new works. (P.12)*
Cyber security	Concerns	*Nothing in the internet world is that extra safe. (P.10)* *Of course, we do not leave personal data where everyone can access it. But we can be more educated on this subject. (P.3)* *Social media is an addiction now. (P.2)* *We can see advertisements about the things we talk about, maybe even think about. (P.5)* *Whether I want it or not, my files and data on my computer can be accessed somehow. For this reason* *I don’t think it’s safe. My thoughts on the security-related part of technology are always an enigma. (P.6)* *During the teleconsultation, you tell your psychiatrist or psychologist your private things. Someone who wants to influence you badly can reach what you speak. (P.12)* *I do not know how this data is used, for what purpose, and where it is transmitted other than where I share it. I don’t know if it is abused or not. I can’t comment on either. (P.12)* *I feel helpless about the Personal Data Protection Law. (P.14)*
	Competence and improvement needs	*I am not fully equipped for the security of personal data. (P.4)* *I know how to choose the right source and who to consult to reach that right source. (P.16)* *People learn a subject best by experience. Events related to the ethical use and safety of this technology in social media should be shown to more people. (P.16)*

AI: Artificial intelligence, P: Participant

Participants thought they had the basic knowledge and competence to fulfill their professional responsibilities in digital literacy. However, a need for development was defined due to different applications and evolutions in this field. One participant stated, “Having digital resources and being able to use them is not sufficient to be digitally literate. We must be able to use, understand and produce information (P.7).”

The students welcomed new technological developments such as robotic surgery, decision support software, and prostheses. One participant said, “If we talk about robotic surgery in the operating room, serious errors can even be reduced to zero. (P.15)” This idea was supported by many participants that problems caused by human errors can be reduced with advanced technology. One participant expresses the possible contribution of decision support systems as follows. “Decision support software takes data many times more than the number of patients physicians can see in their lifetime and draws conclusions that need to be learned. For this reason, this system can see things that a human cannot see (P.3)” Many participants supported the contribution of robotic surgery in solving problems caused by human physical inadequacy and the possibility of decision support systems to reduce errors.

It was observed that the students were concerned that the development of new technologies would negatively affect human relations and interaction. A comment from a student summarized the negative reflection of technology on humanity, “I think interactions involving human relations rather than technology are important in treating, diagnosing, or delivering patient care for most diseases. So maybe we can count this as the difficulty of new technologies (P.15)” A majority of participants expressed their opinion that developments in the field of telemedicine will negatively affect human contact.

Participants perceived the use of new technologies as an area of ​​growth. Some students seemed willing to generate new technologies and said they could quickly adapt to developments. However, costs and limitations in access were seen as obstacles. One participant stated, “New technologies, especially the newest technologies, are very costly in the first place. For this reason, I can’t reach them easily in my private life or school education (P.12).”

The contribution of artificial intelligence in the health field was generally considered positive. The most critical concern in this regard was the changes it would bring in professional practices and the decreased need for labor in some areas. It was stated that people should be trained for newly developing professions. One student emphasized this situation: “Professions may evolve, and new careers will emerge, and as a result, we will need people who have been trained to be able to do these new works (P.12).”

Cybersecurity has been discussed with the most significant concern and participation by the students. The participants voiced their concerns in striking sentences “Nothing in the internet world is that extra safe. (P.10)” and “I feel helpless about the law regulated by the Personal Data Protection Authority (P.14).”

## Discussion

This study aims to assess the digital literacy level and awareness of students studying health-related sciences at the same university and determine if there is a need to reorganize the curriculum accordingly.

Significant gender differences were observed in the participants’ exposure to coding education. Although the student body of this study consists of 77,10% females, a higher percentage of this gender (67.85%) reported not receiving any coding education compared to males (47.71%). On the other hand, more males reported starting coding education in high school and university compared to their female counterparts. However, no notable gender differences were found regarding self-learning proportions.

Gender differences in computational science have been documented before. Studies on kindergarten and primary school-aged children portray boys perform better in coding [24, 25]. Yücel and Rızvanoğlu found that girls showed a lower perception of computer competence and a higher perception of code difficulty compared to boys in middle school children [26]. It was also reported that there was a gender difference in favor of men regarding computer and internet access, frequency of use, and experience in young people [27]. Another study pointed out that while men feel competent in digital technologies, especially in technical and educational modules, women use these technologies more for social purposes [28]. Male students studying Medicine rated their knowledge of electronic information and communication technologies in Medicine and Telemedicine higher than females [29, 30].

Females scored lower in the “hardware” field than men. They felt less confident solving technical problems and needed to gain more familiarity with the essential parts of computer and wireless communications. In a study by Robabi and Arbabisarjou, 79% of the participants were female and stated that most of their students were less knowledgeable about hardware than software [31]. Another study, including medical, pharmacy, and dentistry students, revealed that the computer literacy of male participants was superior to that of females [32].

A few hypotheses regarding girls’ lagging in computer science over boys have been generated. Some reports state that boys are more advantageous in performing better in STEM (Science, technology, engineering, and mathematics ) than girls [33, 34]. It is also believed that the sociocultural perception of “coding is a manly job” limits girls from overcoming this stereotypical categorization and developing an interest in computational sciences [35]. Interestingly, we noticed that although our female participants had less previous coding education than males, their views on the importance of digital literacy education were equal to those of their male counterparts. The analysis revealed no significant differences between genders in their perceptions, indicating a shared belief in the necessity of digital literacy education in health-based sciences.

Based on the results of our study, students of both genders received similar scores in the “network,” “ethics,” and “A.I.” fields. Men felt significantly more competent than women in the “software and multimedia,” “hardware,” and “interest-knowledge” domains, and they held a higher global score. Interestingly men most prominently distanced themselves from women in the “security” area. A study to determine the level of cybersecurity awareness among computer science students found that most students had little or no knowledge and that female students were more likely to be victims of cybersecurity threats [36]. Cybersecurity was among the most discussed interview topics in this study. The students noted that they do not feel secure in the internet world. Even one female student stated feeling helpless about the law regulated by the Personal Data Protection Authority. The fact that students spend more time on the internet and use the Information and Communication Technologies (ICT) more often can make them more vulnerable to cyber threats than other individuals [37–39]. It has been suggested that young people in education, especially female students, should be included in cybersecurity awareness programs to protect themselves [40] and be made aware of using effective and regularly changed passwords to safeguard their accounts or websites [41].

Students’ familiarity with “Network” was the highest of the seven domains, followed by A.I. Students’ scores showed that they confidently use the internet for new information, commonly apply ICT for projects with peers, and communicate with friends and professional contacts via software such as Facebook, Skype, and LinkedIn. Similarly, a computer literacy scale applied to first-year Nursing students yielded the highest score in the network field [42]. Also, medical students’ non-academic computer use, primarily for networking, drew attention, and it was emphasized that the curriculum should be created to encourage their computer use for academic purposes [43]. It was suggested that curriculum developers be aware of students’ entry-level technology skills and create a program according to their needs [44].

Knowledge and awareness of students in “A.I.” was the most discussed domain during the interview. While some students doubted A.I.‘s progress in healthcare and expressed their worries about changes in labor, others held a positive attitude about the future of A.I. Mehta et al., stated that although most medical students are hopeful about A.I.‘s potential to perform various healthcare tasks, they may fear ethical and social topics [45]. Incorporating A.I. ethics into the medical curriculum was previously highlighted [46]. In our study, computer engineering students exhibited lower performance on artificial intelligence-related assessments than other disciplines, most probably due to their requirement for a comprehensive understanding of the delineations that define the utilization and non-utilization of artificial intelligence in medical practice.

The idea expressed by a student that the definition of sufficient digital literacy is to be able to use, understand and produce knowledge received support from other students during the interview. This opinion was consistent with Bulger et al. results [47]. They explained that having access to web pages or a student’s fundamental scientific understanding of how to use sources of information does not represent their knowledge of digital literacy. Also, Ng W articulates students’ interest in educational technologies and explains that it is most unlikely that undergraduate students will intentionally use educational technologies in their regular learning routine unless there is a purpose to do so (e.g., as part of an assignment requirement) [17]. Statistical analysis focusing on changes in overall scores of the digital literacy test’s subtopics with years of university education has shown a weak positive correlation between “ethics”, “ software and multimedia,” and “interest and knowledge.” In addition, as the year has increased, the digital literacy total score also has improved.

A study with a population of undergraduate students of the Educational Science Faculty in Jordan revealed that the students had a medium level of awareness about computer technology ethics, and there were no significant differences in participants’ levels of awareness regarding gender or year [48]. Education about plagiarism among pharmacy and Nursing students showed an improved ability to identify plagiarism [49, 50]. Similarly, the students participating in our study showed that their digital ethics awareness regarding plagiarism, copyright and license, personal data protection, and ethics committee rose as their grade level increased. Our findings support the positive effect of education on digital ethics knowledge.

In a study in Egypt, students were ethically aware of the importance of protecting information privacy showing their general sensitivity to this topic [51]. However, this research indicated that students lack an ethical understanding of the propriety of copyright protection for software. Since most students originated from low-income families, illegally downloaded software was considered “morally” justifiable.

Statistical analysis has also shown that “interest and knowledge” and “software and multimedia” positively correlated with students’ years. Our subjects’ educational program seemed to nurture their interest and adaptation to new technologies and improve their ICT skills. Over the years, they felt confident using the internet for medical information and source verification and were familiar with digital teaching materials, communication platforms, and computer languages. ICT proficiency plays a crucial role in fully benefiting from the e-learning environment [52], which is increasingly used as a tool in education [53]. However, students’ habit of studying only from presentations such as PowerPoint may constitute an obstacle to disciplinary knowledge [54]. Although telemedicine should be included in undergraduate education due to its prevalence, there is a belief that it is inferior to a face-to-face meeting between the healthcare worker and the patient [55, 56]. In our interview, our students also expressed their concerns about this issue.

Considering the overall scores, the years differed the most between the 1st and 4th grades indicating that students felt inadequate about digital literacy in the 1st grade. Mainly first-grade university students must be supported with higher-order digital literacy skills [8].

Our study revealed that no significant differences were found between years in “hardware,” “network,” “security,” and “A.I.” areas, indicating similar performance levels and no improvement across the years in these fields. It is suggested that greater attention must be drawn to the education of digital domains receiving low scores [42]. A study performed on college students in Silicon Valley showed they need to be made knowledgeable of how to protect their data. It also noted that educational institutions need an active approach to improving awareness among college students to increase their knowledge on these issues and how to protect themselves from potential cyber-attacks [57]. Also, students can gain a conceptual understanding of A.I. through a digital literacy course [58]. In addition, the benefits of introducing medical students via a rotation in a simulation lab to innovations, such as robotic surgery, earlier during their education were mentioned [59]. Indeed, our students stated in their interview that they welcome robotic surgery since they reduce human errors and are open to learning new technological developments such as decision support software and prosthesis. Nevertheless, they noted that they could reach personal new technologies only sometimes due to the costs.

The highest scores were obtained by biomedical engineering students, except for “ethics.” On the other hand, medical school students gained the highest scores in this field, surpassing biomedical engineering and computer engineering students, where subjects’ view on citing, digital copyrights, and awareness of the Personal Data Protection Law and Ethics Committee was questioned. Moreover computer engineering students received the lowest score in ethics compared to all other departments. Our findings were in line with another study conducted with engineering and computer science students where “software piracy and copyright” and “misuse of computer resources” was not believed to be “unethical” [60]. It has been stated that there is a need for education about ethical responsibility and judgment in engineering and computer science education [61]. Improvement in ethical perception has been noted after the application of a mandatory ethic workshop for incoming graduate engineering students [62]. That Biomedical engineering students are more exposed to technology may explain why they have yielded the highest scores. Their curriculum contains courses such as programming, technical drawings, and they frequently use specialized software and digital tools. Usually, this group of students is prone to digital literacy before university. Medical school students with the second highest score (calculated when computer engineering students were excluded) in our cohort began to feel more confident in digital literacy as the years progressed. This suggests that these students benefit from elective courses like biostatistics, biomedical engineering, bioinformatics, and computer science.

In this study, the Department of Dietetics and Nutrition students obtained the lowest scores in almost all domains, especially software and multimedia, hardware, ethics, interest, and knowledge. Computer skills gain more importance in dietetics, where more studies show that dieticians and their patients frequently encounter ICT [63]. Embedding ICT in dietetics education supports students’ technological talent and learning skills [64]. Meanwhile, monitoring the nutritional intake of patients with validated computer software shows the potential to be reliable and shorten the duration of dietitians’ interviews [65, 66]. The Nursing students received the second-lowest scores in our cohort. Computer literacy, ICT, and information management skills were found to be relevant to nursing practice [67]. An arrangement with ICT for access to ongoing education and training for nurses and midwives at rural locations was experienced as very useful [68].

The limitation of our study was that female students make up the majority. Furthermore, it was necessary to achieve a more equitable distribution among departments due to the higher participation rates of Nursing students. Students who entered departments such as Biomedical Engineering and Medicine with high scores in the Higher Education Entrance Examination were evaluated together with students who entered other departments with lower scores. This situation may have affected students’ interest, knowledge, and skills in digital literacy.

## Conclusion

In our study, female students had less computer knowledge and previous coding education than male students, although they placed digital literacy equally important. The students from Nursing scored lowest in the digital literacy questionnaire after those from the Nutrition and Dietetics department. The highest scores were obtained by Biomedical Engineering students, followed by the students from the School of Medicine. Participants scored the highest in “network” and “A.I.” and lowest in “interest-knowledge” domains. Although their scores were not included in the statistical calculations and were only used for comparison, the digital ethics scores of the computer engineering faculty students, the most qualified department in digital literacy, remained below average, followed by Biomedical Engineering students.

We concluded that the digital literacy of first-year university students should be assessed because students are subjected to unequal computer science education in middle and high school. An encouraging environment in health-based undergraduate education for digital literacy can be created, especially around female students. Additionally, we suggest redesigning academic programs by opening up more space for digital literacy amid the already busy schedules of healthcare students will help to better prepare them for the age of rapidly changing technological developments; in this array, the weak domains may be supported, and the repetition of the strengths avoided. Also, evaluating them regularly to gauge whether students benefit from the revised curriculum. Thus, it will be possible for health-based university students to gain the digital skills required by today’s clinical settings.

### Electronic supplementary material

Below is the link to the electronic supplementary material.

Supplementary Material 1

Supplementary Material 2

## Data Availability

All data generated or analyzed during this study are included in this published article and its supplementary information files. We confirm that we have read the Journal’s position on issues involved in ethical publication and affirm that this report was written with these guidelines.

## References

[1] Atasoy H, Greenwood BN, McCullough JS (2019). The digitization of patient care: a review of the Effects of Electronic Health Records on Health Care Quality and utilization. Annu Rev Public Health.

[2] Krupinski EA (2015). Improving patient care through medical image perception research. Policy Insights Behav Brain Sci.

[3] McBride S, Tietze M, Robichaux C, Stokes L, Weber E (2018). Identifying and addressing ethical issues with use of electronic health records. Online J Issues Nurs.

[4] The age of opportunity– Future Health Index. report 2020| Philips [Internet]. [Cited 2022 Mar 14]. Available from: https://www.philips.com.sg/a-w/about/news/future-health-index/reports/2020/the-age-of-opportunity.Html.

[5] De la Hoz A, Cubero J, Melo L, Durán-Vinagre MA, Sánchez S (2021). Analysis of Digital Literacy in Health through active University teaching. Int J Environ Res Public Health.

[6] Werner R, Henningsen M, Schmitz R, Guse AH, Augustin M, Gauer T. Digital Health meets Hamburg integrated medical degree program iMED: concept and introduction of the new interdisciplinary 2nd track Digital Health. GMS Journal for Medical Education; 2020. 37.6. 10.3205/zma001354 PMC7672380 33225053

[7] Behrends M, Paulmann V, Koop C, Foadi N, Mikuteit M, Steffens S. Interdisciplinary teaching of digital competencies for undergraduate medical students– experiences of a teaching project by Medical Informatics and Medicine. Public Health and Informatics. IOS Press; 2021. pp. 891–5. 10.3233/SHTI210307 34042802

[8] Öncül G (2021). Defining the need: digital literacy skills for first-year university students. J Appl Res High Educ.

[9] Kennedy DM, Fox R (2013). Digital natives’: an Asian perspective for using learning technologies. Int J Educ Dev Using ICT (IJEDICT).

[10] Grant DM, Malloy AD, Murphy MC (2009). A comparison of student perceptions of their computer skills to their actual abilities. J Inform Technol Education: Res.

[11] Cote T, Milliner B. Japanese university students’ self-assessment and digital literacy test results. In S. Papadima-Sophocleous, L. Bradley& S. Thouësny, editors, CALL communities and culture– short papers from EUROCALL. 2016: 125–131.

[12] Guzmán-Simón F, García-Jiménez E, López-Cobo I (2017). Undergraduate students’ perspectives on digital competence and academic literacy in a Spanish University. Comput Hum Behav.

[13] Sánchez-Cruzado S, Campión R, Sánchez-Compaña MT (2021). Teacher digital literacy: the indisputable challenge after COVID-19. Sustainability.

[14] McCoy LG, Nagaraj S, Morgado F, Harish V, Das S, Celi LA (2020). What do medical students actually need to know about artificial intelligence?. NPJ Digit Med.

[15] Kung TH, Cheatham M, Medenilla A, Sillos C, De Leon L, Elepaño C (2022). Performance of ChatGPT on USMLE: potential for Ai-assisted medical education using large language models. PLoS Digit Health.

[16] Lee H. The rise of ChatGPT: exploring its potential in medical education. Anat Sci Educ, 2023. 10.1002/ase.2270 36916887

[17] Ng W (2012). Can we teach digital natives digital literacy?. Comput Educ.

[18] Ustundag MT, Gunes E, Bahcivan E (2017). Turkish adaptation of digital literacy scale and investigating pre-service science teachers’ digital literacy. J Educ.

[19] Martin A, Grudziecki J, DigEuLit (2006). Concepts and tools for digital literacy development. Innov Teach Learn Inform Comput Sci.

[20] Taylor E (2020). We agree, don’t we? The Delphi Method for Health Environments Research. HERD: Health Environments Research & Design Journal.

[21] R Core Team. R: A language and environment for statistical computing. R Foundation for Statistical Computing, Vienna, Austria. 2022. Available from: URL https://www.R-project.org/.

[22] RStudio Team. RStudio: Integrated Development for R, RStudio, PBC, Boston. MA.2022. Available from: URL http://www.rstudio.com/.

[23] Microsoft Corporation. Microsoft Excel. 2019. Available from https://office.microsoft.com/excel.

[24] Sullivan A, Marina UB (2013). Gender differences in kindergarteners’ robotics and programming achievement. Int J Technol Des Educ.

[25] Sullivan A, Marina UB (2016). Girls, boys, and bots: gender differences in young children’s performance on robotics and programming tasks. J Inform Technol Educ Innovations Pract.

[26] Yücel Y, Rızvanoğlu K (2019). Battling gender stereotypes: a user study of a code-learning game,Code Combat, with middle school children. Comput Hum Behav.

[27] Castro Balsa MJ, Trigo González X. Los Estereotipos De género y las TICs en estudiantes de secundaria. Revista De Estudios E Investigación en Psicología y Educación. Dec. 2017; 144–8.

[28] Grande-de-Prado M, Cañón R, García-Martín S, Cantón I (2020). Digital competence and gender: teachers in training. A case study. Future Internet.

[29] Faihs V, Figalist C, Bossert E, Weimann K, Berberat PO, Wijnen-Meijer M (2022). Medical students and their perceptions of Digital Medicine: a question of gender? Med. Sci Educ.

[30] Haluza D, Wernhart A (2019). Does gender matter? Exploring perceptions regarding health technologies among employees and students at a medical university. In J Med Inform.

[31] Robabi H, Arbabisarjou A (2015). Computer literacy among students of zahedan university of medical sciences. Glob J Health Sci.

[32] Zarei J, Rokhafruz D, Dianat M (2012). The study of computer literacy in students of general medicine at Ahvaz University of Medical sciences (academic year 2009–2010). J Health Adm.

[33] Halpern DF, LaMay ML (2000). The smarter sex: a critical review of sex differences in intelligence. Educ Psychol Rev.

[34] Beilock SL, Gunderson EA, Ramirez G, Levine SC (2010). Female teachers’math anxiety affects girls’ math achievement. Proc Natl Acad Sci U SA.

[35] Master A, Meltzoff AN, Cheryan S (2021). Gender stereotypes about interests start early and cause gender disparities in computer science and engineering. Proc Natl Acad Sci.

[36] Slusky L, Partow-Navid P (2012). Students Information Security practices and Awareness. J Inf Priv Secur.

[37] Jeske D, Van Schaik P (2017). Familiarity with internet threats: beyond awareness. Comput Secur.

[38] Aliyu M, Abdallah NA, Lasisi NA, Diyar D, Zeki AM (2010). Computer Security and Ethics awareness among IIUM students: an empirical study. J Inf Technol.

[39] Öğütçü G, Testik ÖM, Chouseinoglou O (2016). Analysis of personal information security behavior and awareness. Comput Secur.

[40] Adamu AG, Siraj MM, Othman SH (2022). An assessment of cybersecurity awareness level among Northeastern University students in Nigeria. Int J Electr Comput Eng.

[41] Alqahtani MA (2022). Factors affecting cybersecurity awareness among university students. Appl Sci.

[42] Lin TC (2011). A Computer Literacy Scale for Newly Enrolled Nursing College Students. J Nurs Res.

[43] Shaheen MA, Talukder M, Tasnim S (2016). Computer and internet use by the Undergraduate Medical Students of Bangladesh. SE Asian Jnl Med Educ.

[44] Maag MM (2006). Nursing students’ attitudes toward technology: a national study. Nurse Educ.

[45] Mehta N, Harish V, Bilimoria K, Morgado F, Gingsburg S, Law M (2021). Knowledge and attitudes on artificial intelligence in healthcare: a provincial survey study of medical students. MedEdPublish.

[46] Quinn TP, Coghlan S. Readying medical students for medical AI: the need to embed AI ethics education. Arvix. Preprint posted online 7 Sep 2021 arXiv:2109.02866.

[47] Bulger M, Mayer R, E,Metzger MJ. (2014). Knowledge and processes that predict proficiency in digital literacy. Read Writ.2014;27(9): 1567–1583.

[48] Iyadat W, Iyadat Y, Ashour RQ, Khasawneh S (2012). University Students and Ethics of Computer Technology usage: human Resource Development. E-Learning and Digital Media.

[49] DeGeeter M, Harris K, Kehr H, Ford C, Lane DC, Nuzum DS (2014). Pharmacy students’ ability to identify plagiarism after an educational intervention. Am J Pharm Educ.

[50] Eaton SE, Christensen Hughes J (2022). Academic integrity in Canada: historical perspectives and current trends. Academic integrity in Canada: an enduring and essential challenge.

[51] Khalil OEM, Seleim A (2012). Attitudes towards information ethics: a view from Egypt. J Inf Commun Ethics Soc.

[52] Kaware SS, Sain SK (2015). ICT application in education: an overview. Int J Multidiscip Approach Stud.

[53] Naghavi MA (2007). Study of teachers and students’ attitude toward e-learning: surveying in Iran’s e-learning universities. J Res Plan High Educ.

[54] Khoo E, Hight C, Cowie B, Torrens R, Ferrarelli L (2014). Software literacy and student learning in the tertiary environment: PowerPoint and beyond. J Open Flex Distance Learn.

[55] Dadlani A, Bernstein S, Welton R (2023). Assessing Medical Students’ Comfort with Telemedicine. South Med J.

[56] Norwood C, Moghaddam NG, Malins S, Sabin-Farrell R (2018). Working alliance and outcome effectiveness in videoconferencing psychotherapy: a systematic review and noninferiority meta-analysis. Clin Psychol Psychother.

[57] Moallem A. Cyber security awareness among college students. In International conference on applied human factors and ergonomics, 2018: 79–87. Springer, New York.

[58] Kong SC, Cheung WMY, Zhang G (2021). Evaluation of an artificial intelligence literacy course for university students with diverse study backgrounds. Comput Educ Computers and Education: Artificial Intelligence.

[59] Mullens CL, Van Horn AL, Marsh JW, Hogg ME, Thomay AA, Schmidt CR (2021). Development of a Senior Medical Student robotic surgery training Elective. J Med Educ Curric Dev.

[60] Watterson C, Lundqvist K, Quilty J. The ethical understanding of entry level engineering and computer science students. Towards a new future in engineering education, new scenarios that european alliances of tech universities open up. Universitat Politècnica de Catalunya; 2022. 10.5821/conference-9788412322262.1247.

[61] Engineering New Zealand (ENZ)., Code of Ethical Conduct. https://www.engineeringnz.org/resources/code-ethical-conduct.

[62] Trabia M, Longo J, Wainscott S. Training Graduate Engineering Students in Ethics. 2016 ASEE Annual Conference & Exposition Proceedings. ASEE Conferences; 10.18260/p.27072.

[63] Pignone MP, Ammerman A, Fernandez L, Orleans CT, Pender N, Woolf S (2003). Counseling to promote a healthy diet in adults: a summary of the evidence for the U.S. Preventive Services Task Force. Am J Prev Med.

[64] Litchfield RE, Oakland MJ, Anderson JA (2000). Improving dietetics education with interactive communication technology. J Am Diet Assoc.

[65] Rangan AM, O’Connor S, Giannelli V, Yap ML, Tang LM, Roy R (2015). Electronic dietary intake assessment (e-DIA): comparison of a mobile phone digital entry app for dietary data collection with 24-hour dietary recalls. JMIR mHealth and uHealth.

[66] Wada S, Yamamoto E, Kobayashi Y, Otsuki M, Takada, Aoi W (2021). Validation of computer software to estimate dietary intake among patients with type 2 diabetes. J Clin Biochem Nutr.

[67] Chipps J, le Roux L, Agabus J (2022). Nursing informatics skills relevance and competence for final year nursing students. Curationis.

[68] Mills J, Francis K, McLeod M, Al-Motlaq ME (2015). Computer Literacy and Information Retrieval Skills: a rural and remote nursing and midwifery workforce study. Collegian.

