# Supplementary material for: Awareness and level of digital literacy among students receiving health-based education
Source: BMC Med Educ. 2024 Jan 8;24:38. doi: 10.1186/s12909-024-05025-w (PMC10773083; doi:10.1186/s12909-024-05025-w)
Supplement: Supplementary file 1 — Supplementary Material 1 [file 12909_2024_5025_MOESM1_ESM.docx]

**SUPPLEMENTARY DATA**

**Supplementary Table 1:** Correlation of scores with classes

| **Scores** | **p-value** | **Spearman Correlation Coefficient (rho)** |
| --- | --- | --- |
| Software and Multimedia | 0.0008*** | 0.1539 |
| Hardware | 0.1866 | 0.0607 |
| Network | 0.3122 | 0.0464 |
| Ethics | 0.0001*** | 0.1830 |
| Security | 0.7519 | -0.0145 |
| AI | 0.8154 | -0.0107 |
| Interest-Knowledge | 0.0041** | 0.1315 |
| Average Score | 0.0225* | 0.1046 |

*95%, **99%, ***99.9% confidence level for statistically significant correlations

**Supplementary Table 2:** Comparison of computer engineering students scores with other participant scores

| **Numerical Value in Groups** | **p-value** | **Other**  **Departments**  **Mean (SD)** | **Computer**  **Engineering**  **Mean (SD)** | **Other**  **Departments**  **Median (IQR)** | **Computer**  **Engineering**  **Median (IQR)** |
| --- | --- | --- | --- | --- | --- |
| Software and Multimedia | < 0.0001*** | 3.52 (0.62) | 4.05 (0.62) | 3.50 (3.25; 4.00) | 4.00 (3.50; 4.75) |
| Hardware | < 0.0001*** | 3.62 (0.67) | 4.19 (0.71) | 3.67 (3.33; 4.00) | 4.33 (3.67; 5.00) |
| Network | 0.1448 | 3.87 (0.61) | 4.01 (0.57) | 4.00 (3.50; 4.25) | 4.00 (3.75; 4.25) |
| Ethics | 0.0229* | 3.74 (0.67) | 3.46 (0.70) | 3.75 (3.25; 4.19) | 3.50 (3.12; 4.00) |
| Security | 0.0059** | 3.64 (0.78) | 4.00 (0.74) | 3.50 (3.00; 4.00) | 4.00 (3.50; 4.50) |
| AI | 0.2985 | 3.82 (0.66) | 3.73 (0.72) | 4.00 (3.33; 4.33) | 3.67 (3.33; 4.33) |
| Interest-Knowledge | 0.2286 | 3.49 (0.66) | 3.63 (0.62) | 3.50 (3.00; 4.00) | 3.75 (3.25; 4.00) |

*95%, **99%, ***99.9% confidence level for statistically significant differences
