# Supplementary figures and images for: Awareness and level of digital literacy among students receiving health-based education
Source: BMC Med Educ. 2024 Jan 8;24:38. doi: 10.1186/s12909-024-05025-w (PMC10773083; doi:10.1186/s12909-024-05025-w)

Figure S1 - Comparison of Mean scores by department in Each Domain


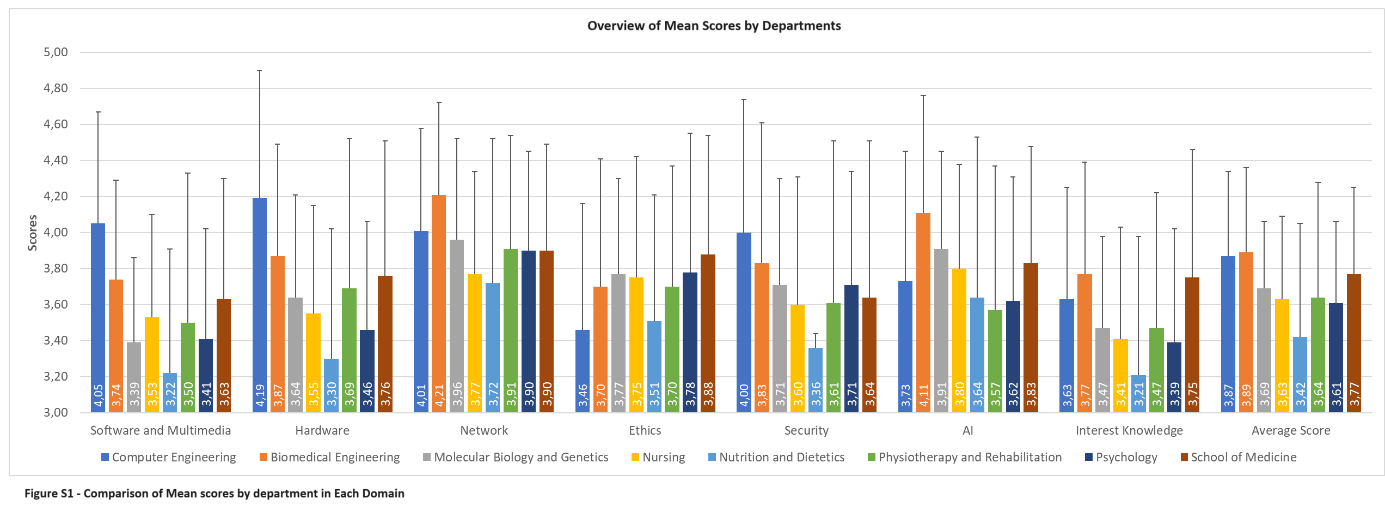

Supplement: Supplementary file 2 — Supplementary Material 2 [file 12909_2024_5025_MOESM2_ESM.docx]
